# The Impact of the Available Infrastructure on the Electric Vehicle Market in Poland and in EU Countries

**DOI:** 10.3390/ijerph192416783

**Published:** 2022-12-14

**Authors:** Karol Tucki, Olga Orynycz, Agnieszka Dudziak

**Affiliations:** 1 Department of Production Engineering, Institute of Mechanical Engineering, Warsaw University of Life Sciences, Nowoursynowska Street 164, 02-787 Warsaw, Poland; 2 Department of Production Management, Faculty of Engineering Management, Bialystok University of Technology, Wiejska Street 45A, 15-351 Bialystok, Poland; 3 Department of Power Engineering and Transportation, Faculty of Production Engineering, University of Life Sciences in Lublin, 20-612 Lublin, Poland

**Keywords:** infrastructure, vehicles, market

## Abstract

The dynamic development of the automotive market entails the need to understand its characteristics. The purpose of this publication is to indicate the changes in the automotive sector resulting from the approaching electromobility. In order to achieve this goal, the market of electric and conventional vehicles in Poland are discussed. This paper discusses issues related to employment in the automotive industry, analyses the location of factories of the largest vehicle manufacturers and analyzes the structure of sales. The development of electromobility in Poland against the background of other EU countries was analyzed in detail. The analysis was based on data from September 2022. The analysis has been conducted by means of computations of the correlation of individual variables (numbers of different types of vehicles: traditional vehicles, BEV and PHEV). The presented results show that the number of BEVs and PHEVs is increasing, and the infra-structure is developing. The number of PHEV cars is usually higher than BEV ones. The results of the analysis of the vehicle market in Poland show occurrence of the variety of vehicle types and variety of the types of vehicle power supplies with alternating current (AC) or direct current (DC). It can be concluded that standardization would be helpful for further development. The number of charging stations is small compared to the number of vehicles, and the advantage of AC stations is evident. This means investing in infrastructure is necessary to enable further development.

## 1. Introduction

One of the priorities of the European Union’s environmental policy is to reduce greenhouse gas emissions [1,2]. Increasingly restrictive legal regulations promote solutions that facilitate the achievement of the assumed goals [3,4,5].

The term electromobility is usually understood as the totality of issues relating to the use of electric vehicles. This term refers to both the technical and operational aspects of electric vehicles, including technology and charging infrastructure. It also covers social, economic and legal issues related to the development, manufacture, purchase and use of electric vehicles. Overcoming social, financial and technical barriers requires coordination at the national, corporate and social level. According to the legal regulations for electromobility and alternative fuels, it is urban mobility that is driven by electric vehicles [6,7]. Therefore, electromobility is part of social mobility, i.e., the mobility of people to achieve their social objectives, in this case with vehicles powered by electricity from mobile sources (batteries). For this reason, the phenomenon of electromobility is closely linked to the energy aspects of transport systems and social policy. European Union environmental legislation can speed up the construction of the necessary infrastructure by obliging project developers and building owners to install charging stations [8,9].

When analyzing the issue of electromobility, it is important to emphasize the importance of aspects related to environmental protection, consumer awareness and knowledge of e-mobility and consumer purchasing behavior [10]. When reviewing the results of scientific work, it is important to note the need to link the results of recent scientific research with an emphasis on issues focusing on the social aspects of electric vehicles and their connection to specific sustainable development goals [11]. Knowledge of electric vehicle perceptions and assessment of their social impact, and the area of user experience and its relationship to sustainability goals enables a better understanding of electromobility [11].

The rapid development of electromobility in the world has been observed for many years. Scientific papers examine regions of the EU, the US, Japan and other parts of the world [12,13]. Each of these regions of the world is different not only from the point of view of the level of economic development, geographic location, level of wealth of citizens access to energy resources, but also from the point of view of the level of awareness of citizens on the impact on the environment and their existing preferences.

Consumer purchasing power and marketing support for electromobility are noticeable [14]. Governments are introducing policy instruments to promote electric vehicles in the form of not only tax and infrastructure rebates, but also financial incentives to purchase such a vehicle [15,16]. The architecture of the market, publicly available charging points, the catalog of incentives and the obligations of public entities in the field of electromobility are analyzed in detail [17]. Expert studies and forecasts are being produced on the formation of the electric car market, its impact on employment, technological progress, aspects of reducing greenhouse gas emissions, the management of energy resources, the formation of consumer attitudes, the development of international trade, the need to change or adapt legal solutions and many other aspects. An important role in the development of electromobility is played by a country’s policies for its development [18,19].

To combat climate change, the European Parliament adopted the European Climate Law, raising the 2030 emission reduction target to at least 55%—“Fit for 55 in 2030”. Achieving climate neutrality is planned by 2050 [20,21].

One of the solutions promoted by the EU is electromobility, which has driven technological development in the automotive industry for several years. Car manufacturers have more electric vehicles in their offers, and with the increase in the number of cars, the public charging infrastructure is also expanding. The development of electromobility is essential for the environment and has a positive impact not only on the automotive industry, but also on the entire economy [22,23,24]. Not only does this sector include the production of electric vehicles, but also the extraction of raw materials, the modernization and development of infrastructure and advanced recycling as part of the circular economy [25,26].

The dynamic increase in the number of electric vehicles predicted by experts will contribute significantly to increased electricity demand in the electricity grid. If the charging patterns of electric vehicles can be correlated with the needs of the electricity system, it is possible to manage these needs through flexibility on the demand side. The issue of flexible energy management is particularly important today, with a precarious market situation where trends in electricity prices are currently very hesitant, with a strong growth trend in the near future.

There are no emission-free cars. Electric cars are locally emission-free. This means that they do not emit any fumes and do not pollute the surrounding air in the place where they are used. The extent to which an electric car is truly emission-free depends on the source of energy used to power the battery.

The development of the level of implementation of electromobility solutions in the individual EU Member States varies [27,28].

Electromobility is one of the fastest developing branches of the Polish economy. According to the source of “Licznik Elektromobilności” (Electromobility Counter), run by PZPM (Polish Association of the Automotive Industry) (PZPM) and PSPM (Polish Association of Alternative Fuels), there were 54,795 electric passenger cars on Polish roads as of the end of September 2022 [29]. Fully electric vehicles (BEV—Battery Electric Vehicles) accounted for approx. 50% (27,200 units) of this part of the vehicle fleet and the remaining part (also approx. 50%) were plug-in hybrids (PHEV—Plug-in Hybrid Electric Vehicles) (27,595 units). The fleet of electric vans and trucks consisted of 2461 units. The fleet of electric mopeds and motorcycles is also growing and included 15,765 units as of the end of September and the number of hybrid cars and vans increased to 439,160 units. At the end of last month, the fleet of electric buses in Poland increased to 788 units.

Parallel to the fleet of electric vehicles, the charging infrastructure is also developing [29]. In addition to building new charging stations, attention must also be paid to their reliability and safety. They must be properly inspected before being put into service and subjected to appropriate regular maintenance and servicing during service. Each charging station must comply with international regulations and have the required certificates (including UL 2202 Electric Vehicle Charging System Equipment and cMETus) [30,31].

At the end of September 2022, there were 2460 generally available charging stations for electric vehicles in Poland (4738 charging outlets). Approximately 28% of them were fast DC charging stations and 72% were slower AC chargers with a power less than or equal to 22 kW. In September 2022, 33 new public charging stations (79 charging outlets) were launched.

Should the experts’ predictions come true, in 2023, every fifth Pole will decide to buy an electric car [32].

The National Reconstruction Plan (NRP), funded by the European Union, will be of great help. The financial support includes investments in transport and electromobility and, thus, in a clean environment. Among other things, the NRP includes investments in electric mobility, the conversion of the urban transport fleet from diesel to electric or hydrogen, and incentives for the purchase of electric cars for individual drivers. The NRP includes proposals that were already adopted in the Electric Mobility Act 2018, long before the outbreak of the pandemic [33].

In addition, according to the Polish NRP, clean traffic zones are established in cities with more than 100,000 inhabitants where air pollution standards are exceeded. According to the Law on Electric Mobility and Alternative Fuels, electric, hydrogen and natural gas cars and combustion cars complying with the highest Euro standards will enter the clean traffic zones, the first of which in Poland will be established in Krakow and Warsaw. The first of these cities will probably do so in 2024 [34].

An electric car consists mostly of the same components as a combustion car. The main difference is in the batteries which are much larger than those of traditional petrol and diesel cars.

Currently, almost all electric vehicles in the world use mainly three types of lithium-ion batteries [35,36]. NMC batteries (with cells based on nickel, manganese and cobalt), NCA batteries (nickel, cobalt and aluminium) and LFP batteries (phosphorus and iron);the first two types are commonly used in passenger electric cars, while LFP cells are used, e.g., in electric buses. Each of these types of batteries uses lithium-ion cells, but contrary to its name, a modern lithium-ion battery contains only 1.5 percent of pure lithium.

The use of e-cars raises other problems, such as the recycling of batteries after their end-of-life. According to Greenpeace, between 2021 and 2030, 12.85 million tons of lithium-ion batteries worldwide will have become unusable in electric vehicles [37]. At the same time, over 10 million tons of lithium, cobalt, nickel and manganese will have been extracted from the ground to produce new batteries. The policy of the European Union in this area promotes such legal solutions that are supposed to force producers to ensure that used batteries do not end up in landfills. Instead, the batteries can be reused in other less demanding applications or recycled.

Currently, there are three main methods of recovering materials from used lithium-ion batteries used in electric cars [38,39]. The most common is pyrometallurgical recycling whereby the valuable metals that are components of lithium-ion batteries are subjected to high-temperature treatment, which allows the used materials to be recovered in the form of an alloy of specific metals. There is also hydrometallurgical recycling, in which the valuable components of the battery are leached, then, through some physical and chemical reactions, they are captured from the resulting solution. Partial recycling is the process of partially recovering materials from the battery, while preserving part of the battery’s cathode and regenerating it.

From the point of view of economic profitability, the sense of recycling is strongly dependent on the prices of metals that are obtained as a result of recycling [40].

There are many studies, reports and expert analyses concerning the analysis and development of the electromobility market in the European Union and in the world, e.g., the EY (Ernst & Young) and Eurelectric report, EV Readiness Index [41,42,43].

According to a report prepared in 2022 jointly by EY and Eurelectric, by 2030 the number of zero-emission vehicles in Europe will have increased from 5 to 65 million, and in the next five years it will have doubled to reach 130 million cars [31]. This creates a challenge for the power grid, as by 2035 it will be necessary to create as many as 65 million charging stations. The report indicates that only by 2035 a total of 65 million charging points will be required, of which more than 9 million will be public stations. According to experts, by 2035, 60% of charging points will be located in the UK, Germany, France, Italy, The Netherlands and Spain. An additional 10% will be located in Poland, Sweden, Portugal and Denmark. As a result, 70% of Europe’s infrastructure will be available in just 10 countries. The report emphasizes that the biggest challenge, however, will not be the energy consumption itself, but the unpredictability in the way electric vehicles is charged.

The EV Readiness Index is a comprehensive analysis that shows the readiness of the European Union countries to transform in the field of transport electrification [43]. The EV Readiness Index evaluates the EU countries based on an analysis of the infrastructure that enables the implementation of electromobility. The state of electromobility in 22 EU countries was analyzed. While assessing the development of the electromobility market and readiness for transport transformation, the ranking took into account factors such as the number of registered cars per capita, the number of charging stations per capita, the percentage of fast chargers per kilometer of motorway, government subsidies for the purchase of electric vehicles, tax exempts and energy costs. According to the 2022 Report, in the case of vehicle ownership costs, Poland showed the largest decrease in the indicator due to the increase in taxes.

In recent years, consumers in Poland and around the world have increasingly used services as part of the so-called sharing economy. It is a new social and economic phenomenon, which requires a shift away from the ownership of goods and resources towards the sharing of goods and resources. The sharing economy covers transport services (Uber, BlaBlaCar, Veturilo), tourism (couchsurfing, Airbnb), financial services (coconut), catering (Quertes), labour market (ShareSpace) and automotive industry (car sharing). According to estimates by PwC (PricewaterhouseCoopers), the global turnover of the sharing economy will reach USD 335 billion in only five key sectors by 2025 [44,45].

The main scientific contribution to this article is to provide an overview of potential types of electric vehicle charging used in Polish conditions, and to indicate the growth trend in this area. Both the number of electric vehicles and the number of charging stations are increasing, which seems to be a natural consequence of the development of the electromobility market. Nevertheless, it should be clearly emphasized that the development of the automotive market in the area of electromobility is slower than economic and political assumptions. The automotive market requires observation and adjustment in the area of production, infrastructure development and a change in the awareness of drivers who are also consumers.

## 2. Materials and Methods

Based on official reports from the field of electromobility and power supply systems in Poland and Europe, the team conducted its own scientific studies. In order to conduct a detailed and up-to-date analysis (as of September 2022), other source studies held by the scientific team were used for in-depth analysis. In the context of the collected source material, a detailed analysis was made of the development of the sector, which covered Poland and Europe [46,47,48,49,50,51].

The manuscript is particularly focused on the case of Poland due to its geographical location and the observed energy transformation and the relatively short membership period among the European Union Member States [52].

The statistical analysis that was conducted to verify the collected data was performed using the Statistica 13.3 SW. For this purpose, an analysis of the correlation of individual variables (different types of vehicles: in particular, traditional vehicles, BEVs and PHEVs to conduct correlation analysis of individual variables.) was conducted. The correlation of linear relationship between the X and Y variables was investigated. Further research was conducted by analyzing the correlation relationship between the studied characteristics. This was done by starting with calculating the correlation between the variables, then drawing a graph. The relationship between variables is shown in graphs, called scatterplots. In the rectangular coordinate system on the abscissa axis, we mark one variable and on the ordinate axis the values of the second variable. The points corresponding to the individual values of the features form a correlation scatter plot. It rarely happens that the selected points lie exactly on a straight line (full correlation), the more common configuration consists of many selected points lying more or less along a specific curve (usually a straight line). As the correlation becomes less and less excellent, then the points begin to disperse and move until they reach a shapeless point cloud (no correlation). Positive correlation occurs when the increase in the value of one feature corresponds to an increase in the mean values of the other feature. The strength of the interdependence of two variables can be expressed numerically by means of many measures. The most popular is the Pearson’s linear correlation coefficient-marked with the symbol r (x, y) and assuming values in the range [−1, 1]. If both variables are measurable and have a distribution close to normal, and the relationship is rectilinear then it gives the possibility to calculate Pearson’s correlation coefficient. Hence the name Pearson’s linear correlation coefficient. When interpreting the Pearson’s linear correlation coefficient, it should be remembered that the value of the coefficient close to zero does not always mean no dependence, but only no linear relationship. The important point is that its absolute value informs about the strength of the relationship, while the sign of the correlation coefficient informs about the direction of the correlation. If r (x, y) = 0, it means a complete lack of correlation between the examined variables X and Y. It is noted that the correlation between variables is stronger the closer the absolute value of the correlation coefficient is to one.

As the analyses interpret, the strongest correlation was noticed between the data on BEV and PHEV vehicles. In addition, the manuscript also performed a correlation analysis of the various variables (i.e., vehicle charging stations—AC and vehicle charging stations—DC).

### 2.1. Employment Scenario in Automotive Industry in Poland and Selected EU Member States

Employment and the level of remuneration for work undoubtedly affect the development of electromobility.

In July 2022, the unemployment rate in the euro zone was 6.6%, compared to 6.7% in in June 2022. The unemployment rate in the EU was 6.0% in July 2022 and 6.1% in June 2022. Poland is among the countries with the lowest unemployment rate. In July it was 2.6% [53].

Positive changes can also be seen in the labour market of people under 25 years old. There was a decrease from 8.6% in June 2022 to 8.4% in July 2022, with an average of 14% youth unemployment in the EU. Eurostat estimates that 12.931 million men and women in the EU, of which 10.925 million in the euro zone countries, were unemployed in June 2022. Compared to the same month a year earlier, unemployment fell by 2.311 million people in the EU and by 1.957 million people in the euro area. In June 2022, 2.546 million people under 25 were unemployed in the EU, including 2.073 million in the euro zone. The youth unemployment rate was 13.6% in both the EU and the euro zone. Compared to June 2021, unemployment in this age group decreased by 527,000 people in the EU and 450,000 in the euro area.

The automotive industry is one of the pillars of the Polish economy. Currently, it accounts for 8% of GDP and about 13.5% of export value. Depending on the scenario, the share of electromobility in Poland’s GDP in 2050 could range from below 3% (passive option) to around 4% (neutral option) to almost 6% (active option). In 2021, almost 439,000 new vehicles were made in Poland [54,55].

**Figure 1:**
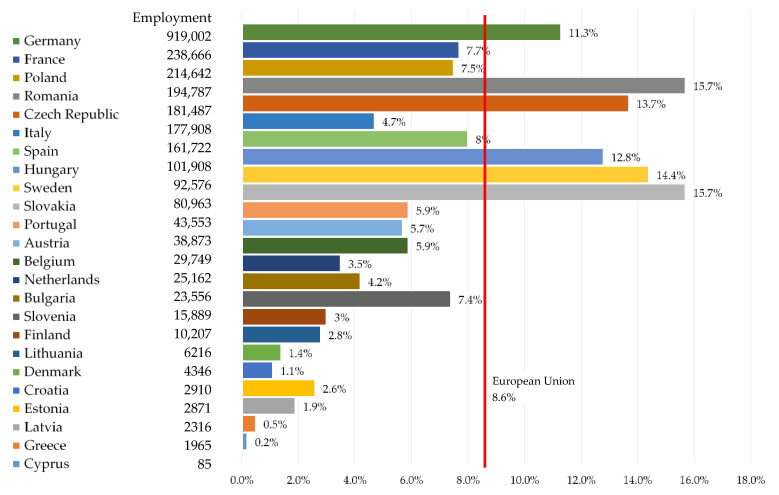
EU employment in the automotive industry [57,58].

Employment in the Polish automotive industry in related industries is about 400,000. people, i.e., 7.6% of all employees in industry. This puts Poland in the third place in the European Union (Figure 1). Most people employed directly in the automotive industry work in Germany—as many as 919,000. France is in second place with 238,000 jobs, and Poland is in third place (214,000). The top five also includes the Czech Republic (181,000) and Romania (194,000). In total, these five countries employ over 1.7 million people, i.e., as much as 67% of all those working directly in automotive factories. There are 2,571,359 people employed in the automotive industry throughout the European Union [56]. The leaders in terms of the number of vehicles produced directly by the automotive industry are the Slovaks (12.8 vehicles per worker), the Slovenes (13.2 vehicles per worker) and the Spaniards (18 vehicles per worker) [57,58].

In Poland, batteries and electric buses are mainly produced. Lithium-ion batteries account for more than 2% of all Polish exports [54,59]. In terms of their production, Poland is the leader in the European Union. Based on BNEF report, on a global scale, Poland ranks the fifth in this respect (first position is China, second is Korea, third is Japan and fourth place is the United States). It should be noted that the BNEF report was prepared in several categories (supply of key raw materials, production of cells and battery components, local demand for EVs and energy storage, industry, innovation and infrastructure and sustainable development) and only in one of these categories did Poland rank fifth. Overall, Poland ranked 14th in the ranking [60,61,62].

Since 2017, Poland has been the leader in the European Union in terms of electric buses manufactured and exported. Between 2017 and 2021, a total of 1937 electric buses were exported from the factories located in Poland to the EU countries. Overall, the sector’s exports in 2017–2021 amount to more than EUR 750 million, i.e., 37.9% of the value of e-bus exports from the European Union [63].

**Figure 2:**
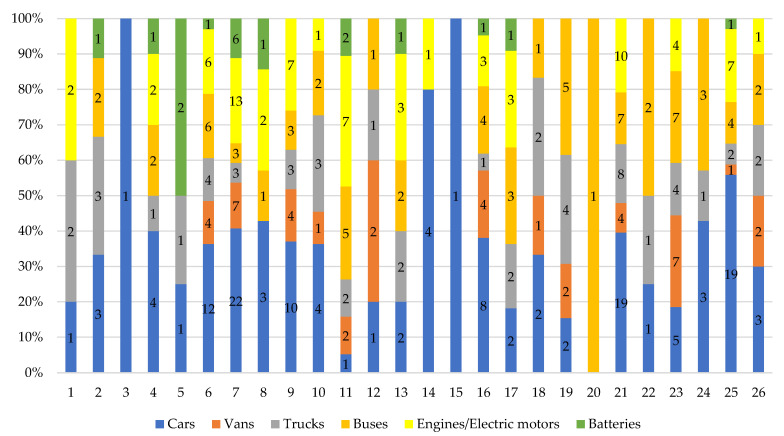
Automobile assembly and production plants in Europe: 1—Austria; 2—Belgium; 3—Croatia; 4—Czech Republic; 5—Finland; 6—France; 7—Germany; 8—Hungary; 9—Italy; 10—The Netherlands; 11—Poland; 12—Portugal; 13—Romania; 14—Slovakia; 15—Slovenia; 16—Spain; 17—Sweden; 18—Belarus; 19—Kazakhstan; 20—North Macedonia; 21—Russia; 22—Serbia; 23—Turkey; 24—Ukraine; 25—United Kingdom; 26—Uzbekistan.

As of 2022, there are 301 car engine assembly and production plants in Europe: 134 factories in Europe produce passenger cars, 41 produce light commercial vehicles (vans), 52 build heavy goods vehicles (trucks), 66 produce buses, 72 make engines and 18 produce batteries (Figure 2). It should be noted that 194 factories are located in the European Union (EU-27). The presented list does not include plants that produce gearboxes, bodywork and any other car parts factories [64].

The drive system of an electric vehicle is responsible for delivering the stored energy in the battery to the engine. The enormous amount of energy that the drive system provides to the motors keeps the electric vehicle efficient and effective. Producing enough batteries can be a problem for many car manufacturers. Below (Table 1) is a list of production locations for electric car components in Europe by major manufacturers [65].

**Figure 3:**
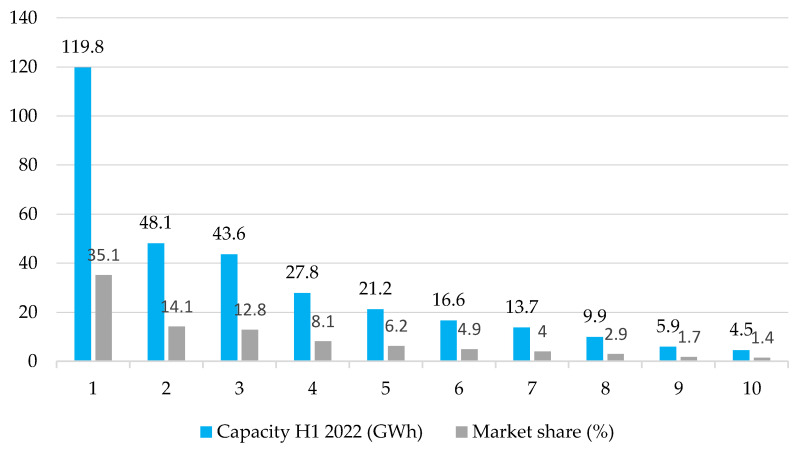
The largest manufacturers of batteries for electric vehicles, January to September 2022: 1—CATL; 2—LGES; 3—BYD; 4—Panasonic; 5—SK on; 6—Samsung SDI; 7—CALB; 8—Gotion Hihg-Tech; 9—Sunwoda; 10—SVOLT.

According to analyses from January to September 2022, batteries for electric vehicles with a total capacity of 341.3 GWh were installed worldwide (Figure 3). The leading provider was CATL with a market share of 119.8 GWh of 35.1%. The second largest manufacturer was LG Energy Solution, which had a market share of 14.1% (48.1 GWh) in the first half of the year. In third place is the former company BYD with a market share of 12.8% (43.6 GWh). Exactly 62% of all batteries for electric cars worldwide came from these three manufacturers [66,67].

The semi-annual summary of the results shows the growth rate of battery production. CATL ended the year 2021 with an output of 96.7 GWh (market share of 32.6%) and reached 119.8 GWh this year. In return, BYD recorded a significant increase in its market share from 8.8% in 2021 (only 6.8% in the first half of 2021) to 12.8% in 2022 [68].

### 2.2. Car Market in Europe

In the first half of 2022, car sales in Europe fell by 13.7%, compared to 2021. However, the share of electric cars in new registrations is constantly growing and diesel cars are decreasing. This may be due to the length of use of the vehicle and the planned ban on registration of combustion cars after 2035. According to the ACEA report, the average age of passenger cars in Europe is 11.5 years. In Poland, 14.1. Slovaks and Latvians (14.0 each, respectively) and Croatians (14.6) have cars of similar age. Slovenians use newer cars—on average 11.7 years old, which is slightly above the European average. Lithuanians (16.8), Estonians (16.7) and Romanians (16.5) drive older cars and these are the highest average ages of passenger cars in Europe. The situation is not much better in Greece—16.0 years [69].

**Figure 4:**
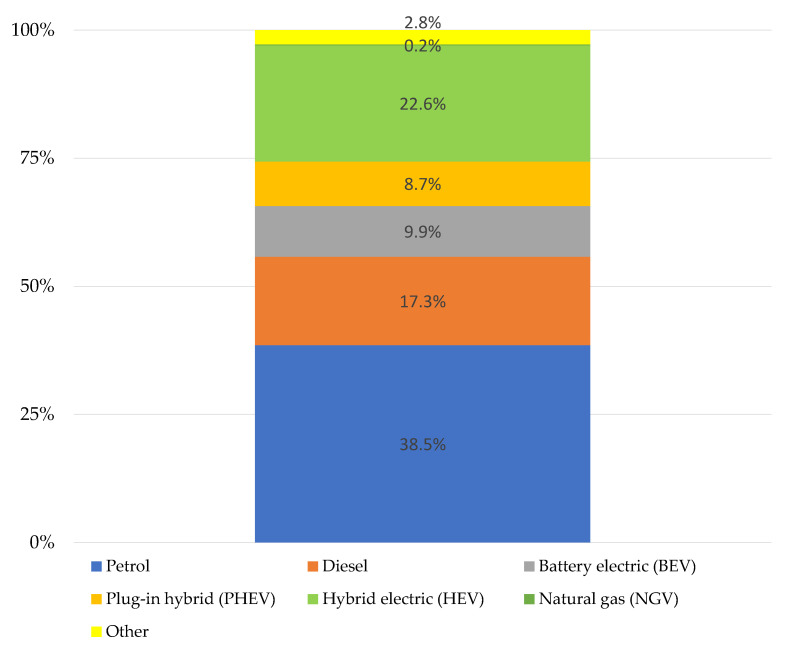
Fuel types of new cars in EU (2022).

In the first half of 2022, sales of battery electric vehicles in the EU accounted for 9.9% of all passenger car registrations (Figure 4). Plug-in hybrid cars accounted for 8.7% of the market share. Cars powered by petrol and diesel oil accounted for 55.8% of the market share (for diesel oil and petrol combined).

**Figure 5:**
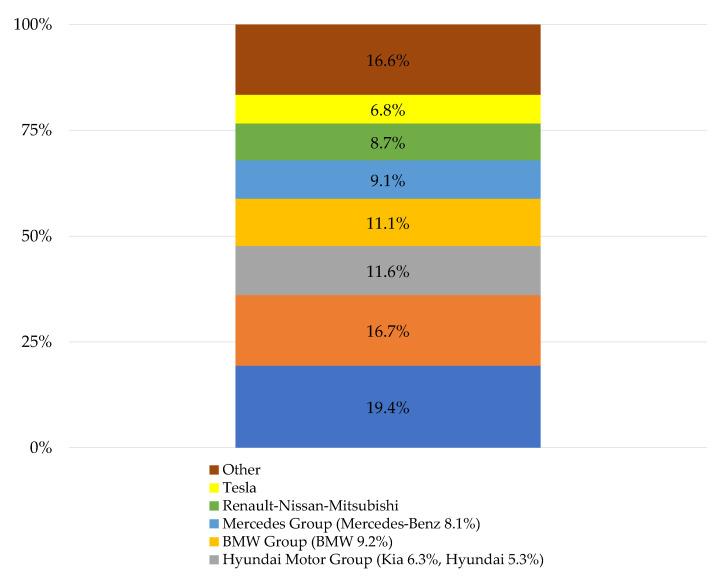
The most popular concerns producing electric cars in Europe (market share in 2022).

From the point of view of the share of the brand and the automotive concern in 2022, the best result on the European market was recorded by the BMW Group (Figure 5) [70].

**Figure 6:**
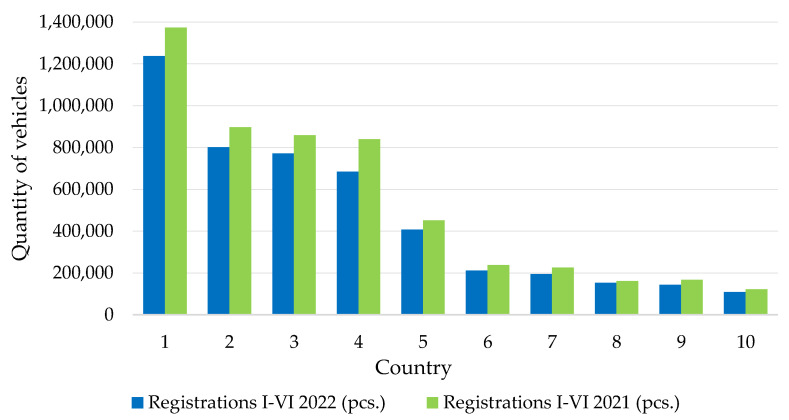
Sales of vehicles in Europe in the first half of 2022: 1—Germany; 2—Great Britain; 3—France; 4—Italy; 5—Spain; 6—Poland; 7—Belgium; 8—The Netherlands; 9—Sweden; 10—Switzerland.

In the six months of 2022, there were 5,027,547 new cars in Europe—13.7% less than in the corresponding period of 2021. In 2021, 5,864,951 units were registered. The largest decreases were recorded in Lithuania (−25.1%) and Italy (−22.7%) (Figure 6) [71].

### 2.3. Electric Car Infrastructure

**Figure 7:**
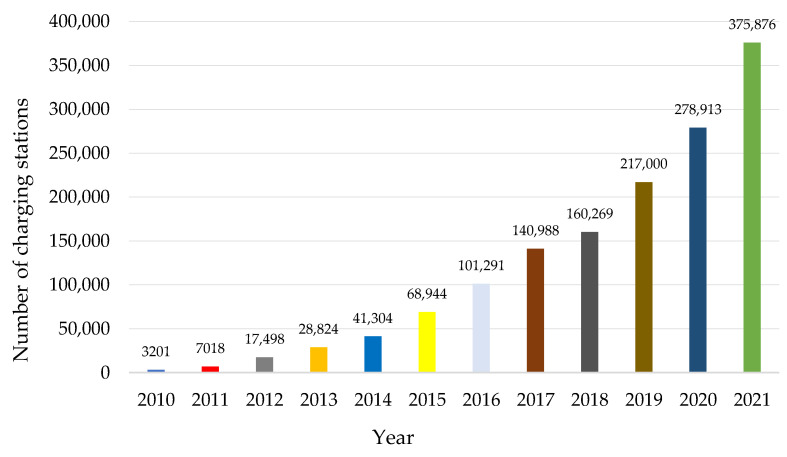
Number of electric vehicle charging stations in Europe 2010–2021.

There are still very few electric car charging stations in Europe. There are far too few of them for the entire European Union to move away from the sale of new internal combustion cars in favor of electric cars in 2035. In 2020, there were over 307,000 charging stations for electric cars in the EU [72]. The differences in individual countries were huge. The distinct leaders in the number of electric charging stations were The Netherlands (90,000 charging points) and Germany (60,000 charging points). They were followed by France (37,000), Sweden (25,000) and Italy (23,500). The fewest charging stations were in Cyprus and Malta, where the number of outlets did not exceed 100. In 2021, there were over 380,000 charging stations across Europe (Figure 7).

### 2.4. Electric Car Infrastructure in Poland

**Figure 8:**
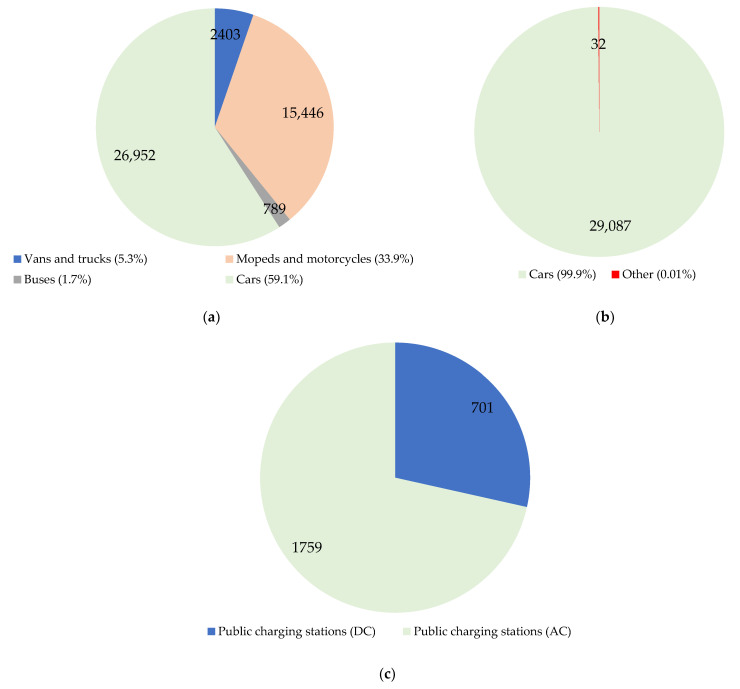
Electromobility in Poland in September 2022: (**a**) BEV all-electric vehicles; (**b**) PHEV plug-in hybrids; (**c**) Charging infrastructure.

In September 2022, there were 58,474 electric vehicles registered in Poland (Figure 8) [73]. Among them, there were 29,355 BEVs (49%) and 29,119 PHEVs (51%). Between January and August 2022, their number increased by 16,139 units, i.e., 44% more than in the corresponding period of 2021. The infrastructure consisted of 2460 public charging stations.

At the end of August 2022, the fleet of electric vans and trucks consisted of 2403 vehicles. The fleet of electric mopeds and motorcycles continued to grow and consisted of 15,446 vehicles. The fleet of electric buses in Poland increased to 789 vehicles.

**Figure 9:**
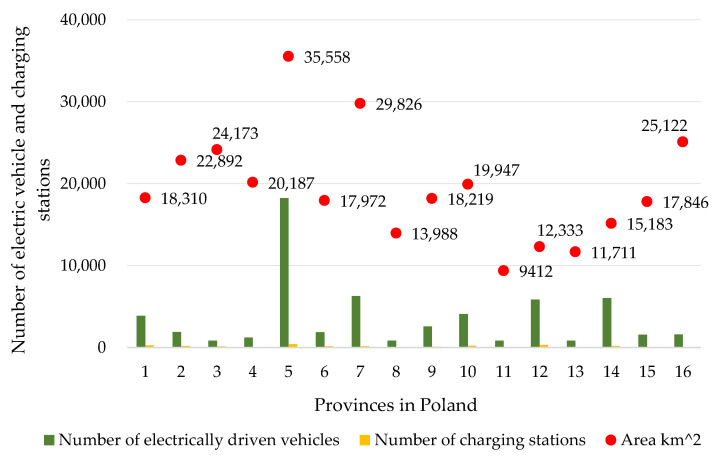
Province area as a function of the number of vehicles and charging stations for electric cars in Poland in September 2022: 1—Pomorskie; 2—Zachodniopomorskie; 3—Warmińsko-mazurskie; 4—Podlaskie; 5—Mazowieckie; 6—Kujawsko-pomorskie; 7—Wielkopolskie; 8—Lubuskie; 9—Łódzkie; 10—Dolnośląskie; 11—Opolskie; 12—Śląskie; 13—Świętokrzyskie; 14—Małopolskie; 15—Podkarpackie; 16—Lubelskie.

A detailed analysis of the development of electromobility in Poland, broken down by individual provinces, is presented in Figure 9 and Table 2. Table 2 presents a summary of the number of vehicles and the number of charging outlets for electric vehicles per province [74,75].

### 2.5. Statistical Analysis

As shown by the statistical test, the null hypothesis H0—Pearson’s linear correlation coefficient is 0, while the alternative hypothesis H1 ≠ 0, i.e., *p* < 0.05—indicates that the value of the correlation coefficient is statistically significantly different from 0. This means that the relationship is significant while the correlation coefficient is not statistically significant [76].

**Figure 10:**
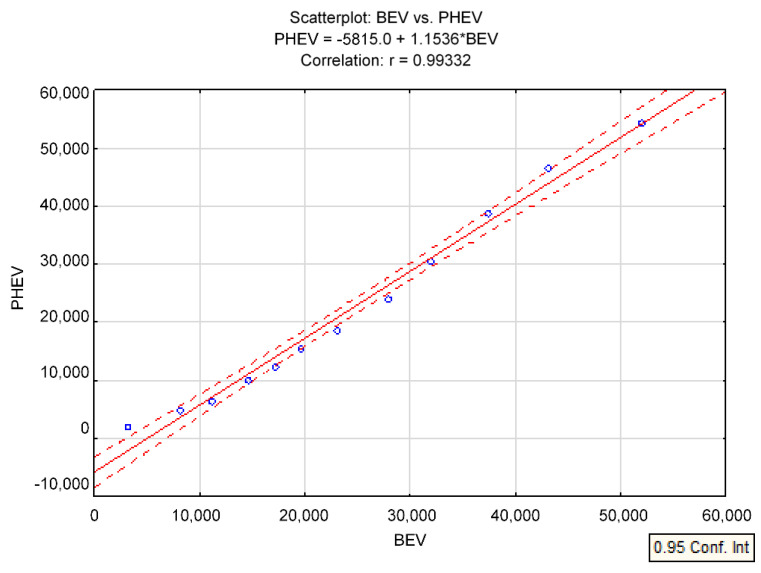
Number of BEVs and PHEVs on the market in Poland in 2019–2021 by linear correlation-scatterplot.

According to the data presented in Table 3, it is noted that there is a strong positive correlation between the number of PHEV (Plug-in Hybrid Electric Vehicle) and BEV (Battery Electric Vehicle). Figure 10 shows the result of the survey, which presents and includes only the variables for these two above relationships.

A summary of the statistical values of each variable is provided in Table 3.

From the study results presented, it is clear that the correlation is strong (r = 0.99332). However, the dependency itself suggests that both electric vehicles (BEV) and hybrid vehicles (PHEV) are becoming increasingly popular.

#### Statistical Analysis—Correlation Analysis between AC and DC Vehicle Charging Stations

**Figure 11:**
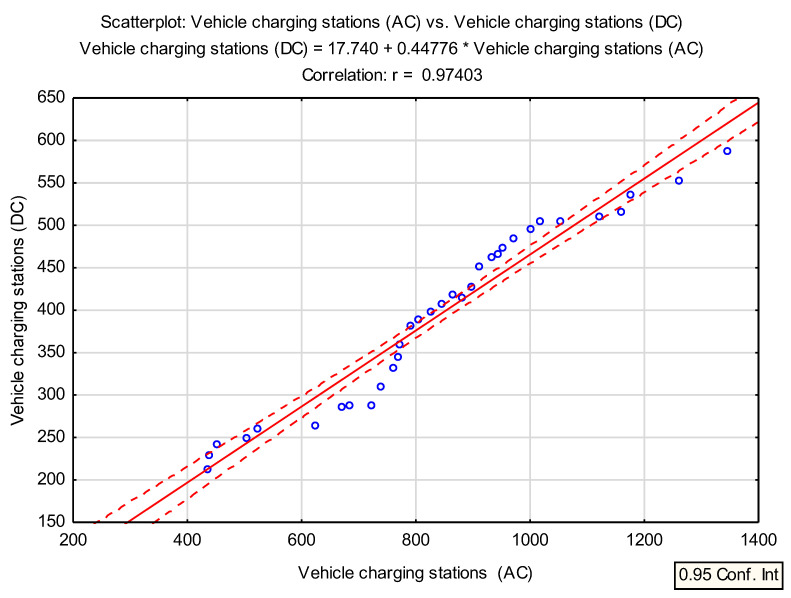
The number of DC charging stations and AC charging stations on the Polish market in the years 2019–2021 in relation to the linear correlation-scatterplot.

In further statistical analysis, a fairly strong correlation was also noticed between AC vehicle charging stations and DC vehicle charging stations (Figure 11). Table 4 shows this relationship, the correlation between individual variables was 0.9740, *p* < 0.05 and amounts to *p* = 0.00.

The correlation between the number of charging stations for DC vehicles and the number of charging stations for AC vehicles for the period 2019–2021 is shown in Figure 11. The analysis presented shows that the correlation is strong (r = 0.97403) and tends to be positive, indicating the great and increasing popularity of charging stations for both DC and AC.

The results of the statistical correlation analysis of the charging station variables AC vs. charging station DC are shown in Table 5.

The individual fields of the results sheet contain arithmetic means of selected variables (843.6970 for vehicle charging stations—AC; and 395.5152 for vehicle charging stations—DC) and the standard deviation values (231.8115 for vehicle charging stations—AC; and 106.5634 for vehicle charging stations—DC). From the data in Table 5, it should be noted that the Pearson correlation coefficient is 0.974033 and the coefficient of determination (r^2^ square of the correlation coefficient) is 0.948739. This is a descriptive measure of accuracy that indicates the fit of the regression to the empirical data. It takes values in the range <0, 1> and reports how much of the total variation in Y observed in the sample was explained (determined) by the regression against the variable X.

The data submitted also show that the larger r^2^ is, the better the dependency and the confidence in a possible regression line is also expected to be greater. The value of the t-statistics, which examines the significance of the correlation coefficient, is, therefore, 23.95313, while the test probability value is *p* = 0.0000, the group size is 33 cases and the linear regression of Y versus X is 17.74039. In addition, the coefficient of linear regression of the variable Y with respect to the variable X is 0.447761 and the intercept linear regression of X with respect to Y is 5.659202, while the coefficient of linear regression of variable X with respect to Y is 2.118851.

The data presented in Table 4 are used to calculate the regression function of variables Y to X and the regression function of variables X to Y. These functions describe the analytical form of the relationship between variables X and Y.

**Figure 12:**
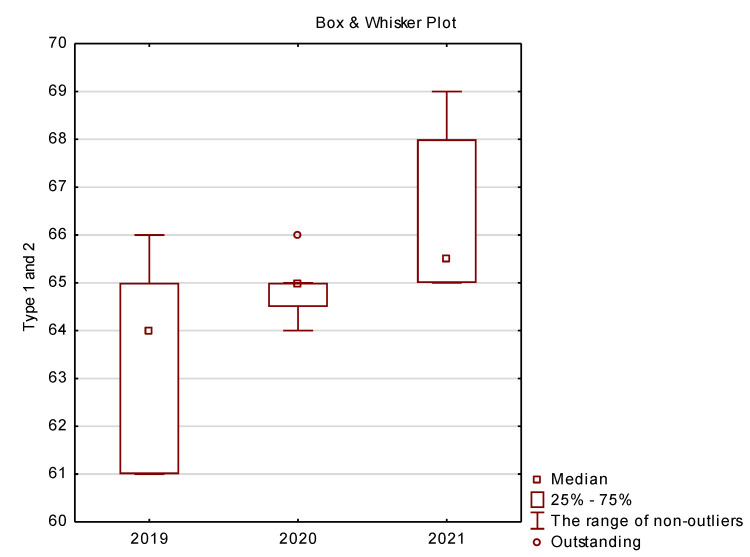
Box plots for the analysis of vehicle charging method—type 1 and 2.

**Figure 13:**
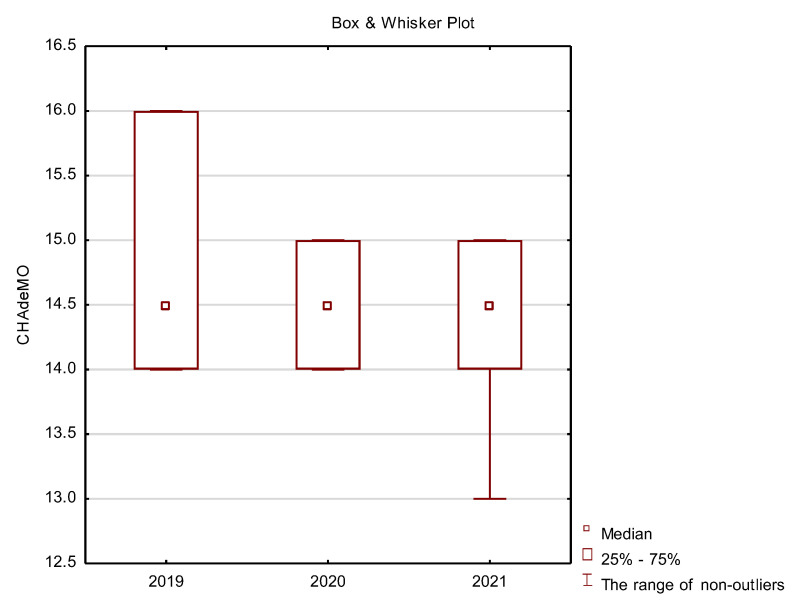
Box plots for the analysis of vehicle charging method—CHAdeMO.

**Figure 14:**
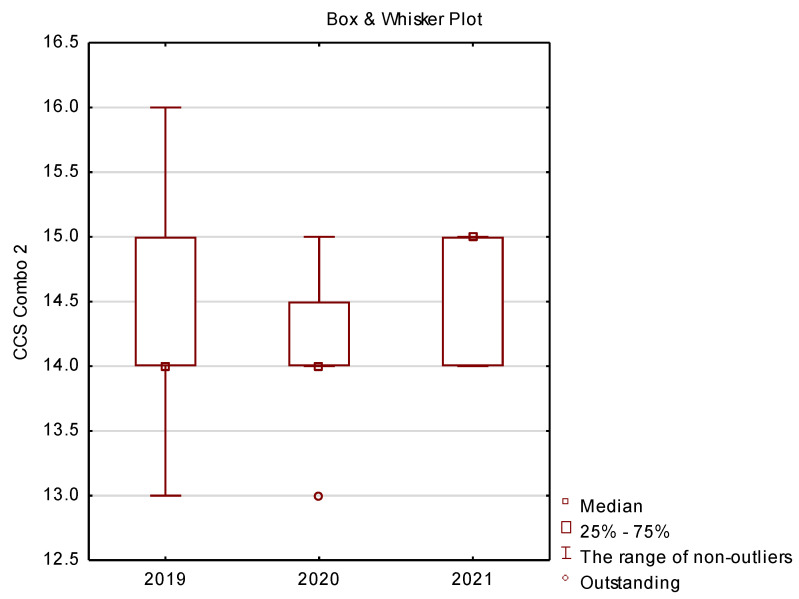
Box plots for the analysis of vehicle charging method—CCS Combo 2.

**Figure 15:**
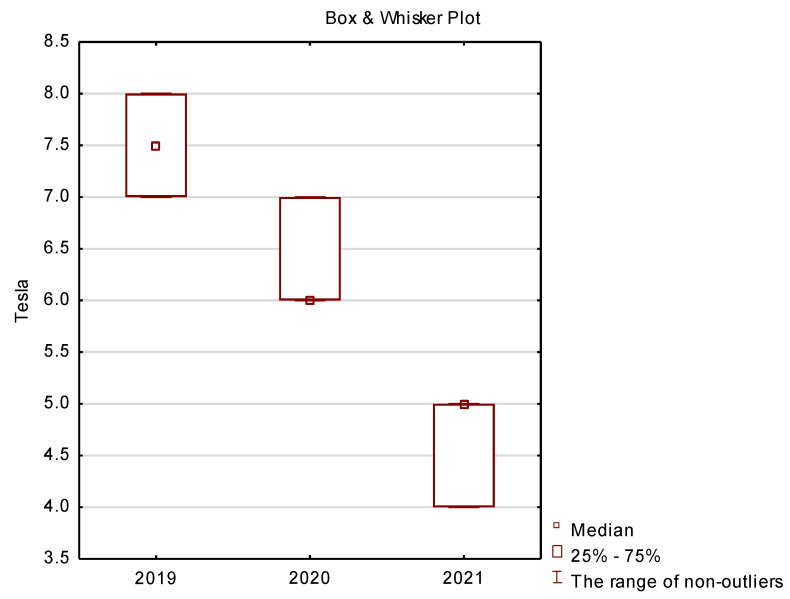
Box plots for the analysis of vehicle charging method—Tesla.

In the final part of this study presents basic descriptive statistics (location measures) comprising median, quartile and minimum and maximum for the following variables: vehicle charging type 1 and 2 (Figure 12), CHAdeMO (Figure 13), CCSCombo2 (Figure 14) and Tesla (Figure 15).

In the case of charging vehicles with TYPE 1 and 2 connectors, which allow charging electric cars with alternating current (AC), it can be seen in Figure 12 that the infrastructure has improved significantly. There is a significant advantage of this type of solutions on the market and there were many of them at the turn of 2019–2021. This is due to the fact that standard, newly produced vehicles have at least one socket that allows them to be charged with alternating current. In the case of some car models, we can also find additional sockets that enable DC charging.

Type 2 plugs (the so-called Mennekes) have become a standard on the European market—the vast majority of currently produced vehicles are equipped with this type of connector. Such plugs have some models of brands such as Mercedes, BMW, Volvo, Tesla and Renault. Of course, there are also electric cars with a type 1 plug on our roads, but they are in the vast minority. Type 1 connector can be found mainly in vehicles of French or Japanese manufacturers. However, in their case, type 2 plugs are becoming increasingly popular.

The CHAdeMO (charge de move) connector was initially used only in cars intended for the Asian market. Over time, it appeared on other continents, mainly due to the popularity of the Nissan Leaf car.

Figure 13 shows that the popularity of this solution in 2021 is lower than in previous years. Kest is a solution especially used in vehicles such as the Nissan Leaf, Nissan e-NV200, Mitsubishi Minicab, Mitsubishi Outlander P-HEV, Kia Soul EV and the American version of the Toyota RAV4.

The standard in Europe today is the extended version of the type 2 connector, i.e., CSS Combo 2, while in the USA the standard is the type 1 extension—CSS Combo 1. CSS Combo 2 differs from the standard type 2 by the presence of two additional risers ensuring faster loading. The CSS Combo 1 solution is popular in cars, such as the Chevrolet Spark, BMW i3 (intended for the US market) and numerous vehicles of other brands intended for the American market. Figure 14 shows that the share of this type of solution in the Polish market is quite stable, or more precisely, the infrastructure in this area changes slightly from year to year.

Due to the fact that the share of Tesla vehicles on the Polish market is not very common, it is also confirmed by the share of Tesla Charging Connector in the market of charging station solutions presented in Figure 15, and it is getting smaller every year. These types of solutions are losing popularity because Tesla Charging Connectors are mainly used in cars such as: Tesla Model 3 (US market), Tesla X (US market) and many other Tesla intended for the US market and, as mentioned in relation to other brands, Tesla vehicles in Poland are relatively much smaller than other brands offering electric cars.

## 3. Results

Electric cars are not a very new technology, but the electromobility sector in the context of private transport is still a novelty on the market. As a result, the prices of this type of vehicle are relatively high compared to cars with conventional drives. The overriding problem is the access to infrastructure. There are still no economically viable methods to recycle the residues from electric cars.

Based on the analysis conducted, it can be concluded that:

1. At the end of 2021, there were 2,571,359 people employed in the automotive industry in the European Union, of which, 214,642 in Poland. Most people were employed in Germany—919,002 people.
2. In the European Union and in Poland, the entire automotive sector is developing very dynamically. It is a significant employer and one of the largest branches of the economy. Currently, the automotive industry in Poland accounts for 8% of GDP and approx. 13.5% of export value. The automotive industry is currently undergoing the fourth industrial revolution. Technologies that help optimize production and artificial intelligence which drives the development of autonomous cars, have a key impact on the industry.
3. In 2022, there are 301 car engine assembly and production plants operating in Europe, of which 194 are located in the European Union (EU-27). Most factories are located in Germany. Among the countries outside the EU, Russia should be mentioned with 33 factories producing cars and components.
4. In the first half of 2022, 5,027,547 new cars appeared in Europe, 13.7% less than in the same period of 2021. Sales of battery electric vehicles in the EU accounted for 9.9% of all passenger car registrations.
5. In 2021, across Europe, there were over 380,000 charging outlets.
6. In September 2022, there were 58,474 electric vehicles registered in Poland, including BEV (49%) and PHEV (51%). The fleet of electric buses in Poland has increased to 789 units.
7. At the end of September 2022, there were 2460 charging stations in Poland for 58,474 electric vehicles.

## 4. Conclusions

The future of electric and hybrid vehicles depends on many factors, including from technological progress, adaptation of road infrastructure and improvement of the methods of obtaining electricity [77]. Most likely, in the near future, we will see an increase in vehicles of this type in our everyday life. In today’s world, we can meet many different threats, ecological, economic, but also social challenges. This results in the introduction of various solutions aimed at keeping up with the changing reality. By definition, electromobility was to be a response to a number of ecological challenges, including environmental pollution, smog and noise [78]. The introduction of electric vehicles to the market may contribute to the reduction in the emission of harmful substances into the environment and to the further development of the vehicle structure and the extension of the areas of their application. As a result of reducing the noise of traffic, the comfort of people staying in areas with increased traffic will improve. However, “quiet” vehicles may turn out to be a certain danger for pedestrians (especially within pedestrian crossings) who are used to the sounds of moving internal combustion vehicles, often much louder than electric ones.

Taking into account the current trends and conditions of vehicle use, in the future we can expect faster development of technologies related to their electric propulsion [79]. The parallel development of all industries related to electric vehicles may turn out to be important: from the methods of obtaining electricity, though, among others, methods of producing batteries and other vehicle components, activities of repair shops, ending with recycling. Importantly, investments in road infrastructure by building more charging points for vehicle batteries are very important. Without it, the development and availability of electric vehicles is impossible. The increase in the use of electromobiles on public roads would also be fostered by the support of their users by the authorities governing the state in the form of, for example, tax reductions. Some countries belonging to the European Union currently use this type of solution, which results in a visible increase in the number of electric vehicles used.

One more important issue to be solved is the problem of energy storage and the capacity of the batteries that serve this purpose.

The cheapest way to charge an electric car is to have your own charger and a properly selected photovoltaic installation [80]. By connecting an electric car to charging in the morning and evening, while the photovoltaic is still producing energy, you can increase self-consumption of energy. Then, the excess energy from photovoltaics is used 100%, while the car always has charged batteries. Currently available electric cars have very capacious batteries, the most common are batteries with a capacity of 20 kW to 50 kW. Therefore, an electric car can be used as an energy store for the home. Cars equipped with the V2H function, i.e., Vehicle to Home, can be charged, then returned energy when it is needed. Special two-way electric car chargers are indispensable for this. Such energy storage in a car may be the answer to the summer overproduction of electricity. In this case, the electric car can partially replace the stationary energy storage. It is enough to connect the car to charging to recharge the battery, then at night the electricity can be taken from the batteries, and in the morning, when there would be a lot of energy production from photovoltaics, the loss will be replaced. However, the energy storage in the car, despite its very large capacity, is not able to 100% replace traditional energy storage. The greatest production of electricity from photovoltaics occurs when most users are away from home (e.g., at work). The use of an electric car for energy storage may, however, become an interesting idea to increase the self-consumption of energy produced by photovoltaics and thus further accelerate the return on investment in both the electric car and photovoltaics [81,82].

The electric vehicle market is likely to develop dynamically in the coming years, which is confirmed by numerous observations and studies. Nevertheless, it is necessary to observe the situation and analyze its potential, because these are huge industrial and infrastructure investments, so in order for the solutions to be widely used, they must be conscious and thoughtful. In addition, the benefits must compensate for the costs incurred and there are many of these in the area of electromobility development.

## Figures and Tables

**Table 1:** Main electric car component manufacturers in Europe.

	Manufacturer																									
Country	BMW	BorgWarner	Bosch	Contemporary Amperex Technology (CATL)	Daimler-ACCUMOTIVE	Daimler	Ferrari	Fiat Chrysler	Ford of Europe	Hyundai-Kia	Jaguar Land Rover (Tata)	LG Chem	Magna Powertrain	Magna Steyr	Northvolt	PSA/Peugeot Citroen	Renault-Nissan-AvtoVAZ	Samsung SDI	SK Innovation	StreetScooter	Thunder Power	Toyota	VDL (Formerly Mitsubishi/NedCar)	VW Group	ZF Friedrichshafen	Zhejiang Geely (Volvo/London Electric Vehicle Company)
Austria													x	x												
Belgium																					x			x		x
Czech Republic																								x		
France						x										x	x									
Germany	x	x	x	x	x	x			x							x				x		x		x	x	
Hungary																		x	x					x		
Italy							x	x																		
The Netherlands																						x	x			
Poland						x						x										x				
Romania									x																	
Serbia																	x								x	
Slovakia									x	x							x							x		
Spain						x										x								x		
Sweden		x													x	x										x
Turkey									x								x					x				
UK	x										x					x	x					x				x

**Table 2:** Detailed list of the number of vehicles and the number of charging outlets in individual Polish provinces in September 2022.

Province	Area km^2^	Number of Electrically Driven Vehicles			Number of Charging Stations		
		Sum	BEV	PHEV	Sum	AC	DC
Pomorskie	18,310	3885	1874	2011	263	198	65
Zachodniopomorskie	22,892	1893	845	1048	190	164	26
Warmińsko-mazurskie	24,173	836	381	455	139	126	13
Podlaskie	20,187	1214	632	582	52	32	20
Mazowieckie	35,558	18,237	9139	9098	413	326	87
Kujawsko-pomorskie	17,972	1883	805	1078	153	106	47
Wielkopolskie	29,826	6280	3186	3094	158	95	63
Lubuskie	13,988	848	423	425	51	27	24
Łódzkie	18,219	2581	1290	1291	109	44	65
Dolnośląskie	19,947	4089	1996	2093	224	164	60
Opolskie	9412	827	420	407	59	41	18
Śląskie	12,333	5856	2834	3022	323	233	90
Świętokrzyskie	11,711	846	488	358	31	24	7
Małopolskie	15,183	6032	3464	2568	197	126	71
Podkarpackie	17,846	1577	755	822	61	32	29
Lubelskie	25,122	1590	823	767	37	21	16

**Table 3:** Statistical values of individual variables in the overall summary.

Variable	Mean	Standard Deviation	Traditional Vehicles	PHEV	BEV
Traditional vehicles	119,216	20,896.59	1	−0.430761	−0.455769
PHEV	22,004.2	17,184.83	−0.430761	1	0.99332
BEV	24,115.6	14,797.29	−0.455769	0.99332	1

**Table 4:** Correlation relationship of AC and DC variables for *p* < 0.05000, N = 33.

Variable	Y-Vehicle Charging Stations—DC
X-Vehicle charging stations—AC	0.974
	*p* = 0.00

**Table 5:** Results of statistical analysis of correlation of variables for vehicle charging stations—AC and vehicle charging stations—DC.

Variable	Mean	Std. Dev.	r (X, Y)	r^2^	t	*p*	N	Constant Dep: Y	Slope Dep: Y	Constant Dep: X	Slope Dep: Y
**X**	843.6970	231.8115									
**Y**	395.5152	106.5634	0.974033	0.948739	23.95313	0.0000	33	17.74039	0.447761	5.659202	2.118851

Abbreviations: **X**-Vehicle charging stations—AC; **Y**-Vehicle charging stations—DC.

## Data Availability

The study did not report any data.
